# Popliteal Artery Injuries in Orthopedic Knee Surgery: A Narrative Review with an Italian Medico-Legal Perspective

**DOI:** 10.3390/diseases14090335

**Published:** 2026-09-13

**Authors:** Giulia Colonna, Giulio Nittari, Taurino Francesco, Pasqualino Sirignano, Giovanna Ricci, Paolo Bailo

**Affiliations:** 1 Vascular and Endovascular Surgery Unit, Sant’Andrea Hospital of Rome, Department of Molecular and Clinical Medicine, “Sapienza” University of Rome, 00185 Rome, Italy; 2 Telemedicine and Telepharmacy Centre, School of Medicinal and Health Products Sciences, University of Camerino, 62032 Camerino, Italy; giulio.nittari@unicam.it; 3 San Camillo de Lellis Hospital, 02100 Rieti, Italy; taurino92@hotmail.it; 4 Vascular and Endovascular Surgery Unit, Sant’Andrea Hospital of Rome, Department of General and Specialistic Surgery, “Sapienza” University of Rome, 00185 Rome, Italy; pasqualino.sirignano@uniroma1.it; 5 Section of Legal Medicine, School of Law, University of Camerino, 62032 Camerino, Italy; giovanna.ricci@unicam.it (G.R.); paolo.bailo@unicam.it (P.B.)

**Keywords:** popliteal artery injury, orthopedic surgery, vascular injury, medico-legal appraisal, healthcare liability, informed consent

## Abstract

Objectives: To provide a narrative overview of popliteal artery injury (PAI) associated with orthopedic knee surgery, focusing on its mechanisms, risk factors, diagnostic challenges, treatment strategies, and medico-legal appraisal within the Italian civil healthcare liability framework. Methods: A non-systematic literature review was conducted using PubMed, Scopus, and Web of Science, including articles published up to December 2025. Original studies, reviews, case series, and case reports addressing vascular anatomy, mechanisms of injury, diagnosis, treatment, and outcomes were selected. During revision, a separate targeted medico-legal search was conducted in PubMed, Scopus, and Web of Science on 7 September 2026, and Italian statutory, judicial, and deontological sources were examined for the civil healthcare liability analysis. Results: The clinical synthesis included 47 studies. Popliteal artery injury was identified as a rare but serious complication of procedures such as total knee arthroplasty, ligament reconstruction, arthroscopy, osteotomies, and hardware removal. Injury mechanisms included thrombosis, arterial incision, pseudoaneurysm formation, and arteriovenous fistulae. Computed tomography angiography emerged as the preferred imaging modality in uncertain cases, while both open surgical and endovascular treatments showed satisfactory results when performed promptly. Delayed diagnosis was consistently associated with worse functional outcomes and increased risk of limb loss. The targeted medico-legal search found only sparse literature directly addressing PAI; the Italian analysis therefore focused on professional conduct, causal attribution, informed consent, and evidentiary reconstruction rather than treating the adverse event itself as evidence of liability. Conclusions: Although uncommon, PAI remains a potentially limb-threatening complication of knee surgery. Thorough anatomical knowledge, early recognition, and timely vascular intervention remain central to clinical management. Within the Italian civil liability framework, the occurrence of a recognized complication neither establishes nor excludes negligence. Medico-legal appraisal should distinguish the primary vascular event from any additional harm attributable to delayed recognition or treatment and should address informed consent and documentation as separate issues.

## 1. Introduction

Vascular complications in orthopedic surgery are rare but potentially serious events. Their incidence is estimated to range from 0.005% to 0.5% [1].

Arterial and venous injuries may present as incisions, pseudoaneurysms, thrombosis, or arteriovenous fistulae and are generally categorized as either indirect or direct. While indirect complications, such as deep vein thrombosis (DVT), are not primarily caused by the surgical procedure itself [2], direct injuries result directly from the surgical intervention and are often linked to factors such as improper placement of retractors, direct vessel trauma, extreme joint manipulation, implant or fixation device malpositioning, joint dislocations, prosthetic loosening, or periarticular ossification [3].

Procedures most frequently linked to vascular injuries include knee arthroplasty, followed by hip arthroplasty, spinal surgery, and knee arthroscopy. These injuries primarily involve the popliteal artery, with the femoral, iliac, superficial, and tibial arteries affected less commonly [4]. Injury to the popliteal artery can cause limb ischemia, tissue necrosis, and potentially lead to amputation. The risk of amputation significantly increases if the injury goes undetected and revascularization is delayed for as little as six hours [5].

Despite its clinical relevance, the current understanding of popliteal artery injury in orthopedic knee surgery remains fragmented. Significant heterogeneity exists in the reported mechanisms of injury across different procedures, early diagnosis and treatment.

### Popliteal Artery Anatomy

The popliteal artery (PA) is the continuation of the superficial femoral artery and represents the main arterial supply to the distal lower extremity. After passing through the adductor canal and the adductor hiatus, it enters the popliteal fossa, where it runs posteriorly to the knee joint.

Along its course, the PA is accompanied laterally by the popliteal vein and gives rise to several genicular branches [6]. Distally, the PA courses along the medial border of the popliteus muscle and divides into its two terminal branches: the anterior tibial artery (ATA) and the posterior tibial artery (PTA). The peroneal artery (PEA) originates from the PTA approximately 2.5 cm distal to the PA bifurcation [7]. In some cases, this division may occur more proximally, with the anterior tibial artery occasionally descending along the popliteus muscle [8,9].

At the level of the knee joint line, the PA is typically located just posterior to the posterior horn of the lateral meniscus, with a mean minimum distance of approximately 1.01 cm (1.06 cm in males and 0.88 cm in females [7,10]. This close anatomical relationship makes the artery particularly vulnerable during surgical procedures involving the posterior aspect of the knee. Therefore, a precise understanding of its anatomical course is essential to prevent intraoperative injury and to allow early recognition of vascular complications.

**Figure 1:**
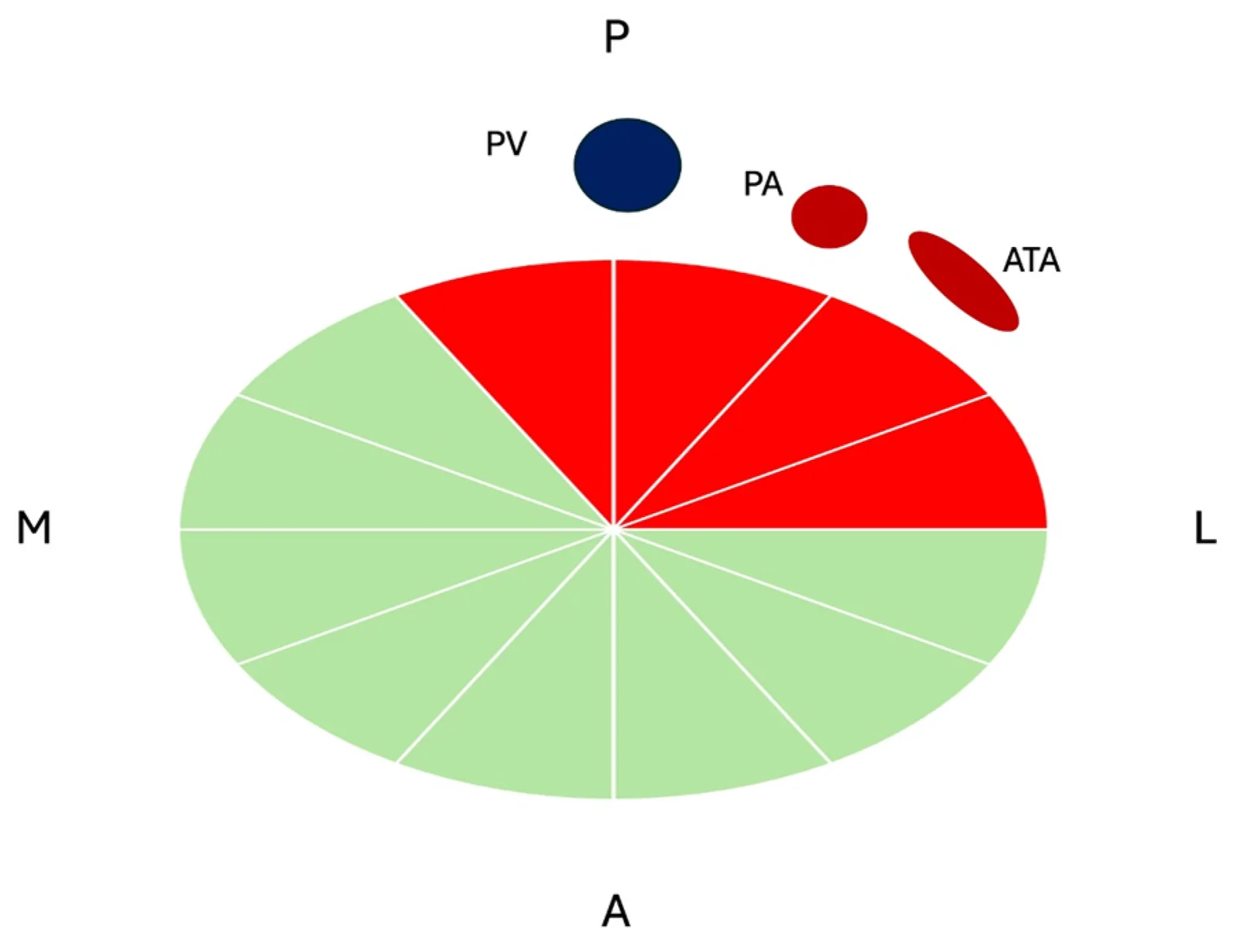
Illustration of the left tibial plateau demonstrating the ‘clock face’. The ‘danger zone’ is represented by the red area between 11.00 and 15:00 o’clock. Modified from Rubash HE et al. Clin Ortohp Relat Res. 1993 Jan (258) 56–63 [11]. Abbreviations: M Medial, L Lateral, A Anterior, P Posterior, PV: Popliteal vein, PA: Popliteal artery, ATA: Anterior tibial artery.

To better visualize the relative positions of these structures, Rubash et al. compared the tibial surface to a clock face as shown for the left knee. The popliteal vein is at 00.00 h, popliteal artery at 01.00 h and the anterior tibial artery at 02.00 h. A ‘danger zone’ between 11.00 and 03.00 h is identified within which deep dissection, the insertion of retractors and screw penetration should be avoided [11,12] (Figure 1).

The risk of iatrogenic injury to the popliteal artery or its terminal branches may be further increased by anatomical variations, which are not uncommon [7]. Consequently, a detailed understanding of the artery’s course, and careful preoperative evaluation of patient-specific vascular anatomy is essential to minimize complications during posterior knee surgeries.

This narrative review aims to address these critical issues by synthesizing the current evidence on popliteal artery injury during orthopedic knee surgery, with particular emphasis on mechanisms of injury, diagnostic challenges, and treatment strategies, and by providing a separate medico-legal appraisal within the Italian civil healthcare liability framework.

## 2. Materials and Methods

### 2.1. Clinical Literature Search

A non-systematic (narrative) literature search was performed using major electronic databases, including PubMed, Scopus, and Web of Science, covering articles published up to December 2025. The search strategy combined keywords and Medical Subject Headings (MeSH) terms such as “popliteal artery injury,” “knee surgery,” “total knee arthroplasty,” “arthroscopy,” “vascular complications,” using different Boolean combinations to maximize sensitivity.

The selection process was based on relevance to the predefined thematic domains of the review rather than on formal systematic eligibility criteria. These domains included vascular anatomy and risk factors, mechanisms and types of popliteal artery injury, orthopedic procedures associated with vascular complications, diagnostic approaches, treatment strategies and clinical outcomes. Titles and abstracts were initially screened to identify potentially relevant publications, followed by full-text assessment when appropriate. Additional articles were identified through manual screening of the reference lists of selected papers, and duplicates were removed.

Eligible publications included original research articles, narrative and systematic reviews, case series, and case reports written in English.

This broad inclusion strategy was considered appropriate because popliteal artery injury following orthopedic knee surgery is a rare complication and the available literature is highly heterogeneous, with a substantial proportion of the evidence derived from observational studies and case-based reports. As the purpose of this narrative review was to provide a broad clinical overview rather than to estimate pooled treatment effects or compare interventions quantitatively, no formal hierarchy of evidence or quantitative quality assessment was applied. Following this process, 47 publications were considered relevant to the objectives of the review and were included in the narrative synthesis.

### 2.2. Medico-Legal Literature and Legal Sources

To address the medico-legal component, a separate targeted narrative search was conducted on 7 September 2026 in PubMed, Scopus, and Web of Science. Two complementary searches were used: a PAI-specific strategy combining popliteal vascular injury and knee-surgery terms with medico-legal concepts, and a broader orthopedic/vascular strategy intended to identify contextual evidence on malpractice, informed consent, diagnostic or treatment delay, documentation, and causal attribution. The exact database strings and raw yields are reported in Supplementary Table S1. The broader search was intentionally sensitive and was used for targeted source identification within the narrative design rather than as an exhaustively screened systematic litigation corpus.

Because legal standards and evidentiary rules are jurisdiction-dependent, the legal analysis was explicitly limited to Italian civil healthcare liability. Primary legal sources were examined separately from the biomedical literature. Statutory provisions were checked through Gazzetta Ufficiale and Normattiva; relevant Court of Cassation decisions and institutional materials were used for civil liability, burden of proof, causation, and consent; and the Italian Code of Medical Deontology was consulted through FNOMCeO for professional duties concerning information and clinical documentation. Clinical publications were used to establish clinical premises, whereas legal propositions were anchored to Italian authorities. Foreign litigation studies were retained only as contextual empirical evidence and were not used to define Italian legal standards. Criminal and disciplinary liability were outside the scope; deontological provisions were used only as professional normative sources.

No single quality hierarchy was applied across clinical studies, litigation datasets, and legal sources because these evidence classes serve different functions. Particular attention was paid to directness to PAI, jurisdiction, temporal applicability, source authority, and the distinction between reported claims, compensation or settlement data, and adjudicated findings.

## 3. Results

The Results section is organized to summarize the main findings of these studies, and includes a brief overview of the anatomy of the popliteal artery, a description of the different types of injury, the orthopedic knee procedures most commonly associated with vascular damage, and, finally, the principal diagnostic approaches and available treatment options are presented.

### 3.1. Type of Damage

Iatrogenic injuries to the popliteal artery during orthopedic knee surgery vary according to the underlying mechanism and the type of damage. These injuries can be classified into arterial occlusion or incision. Thrombotic occlusion typically results from indirect mechanisms, such as joint manipulation or tourniquet application, and presents with limb ischemia. Arterial incision or transection originates from a direct penetrating injury and may manifest as intraoperative haemorrhage, pseudoaneurysm, arteriovenous fistula, or recurrent hemarthrosis [13].

It is worth mentioning that, in rare cases, acute limb ischemia following knee surgery may result from acute arterial spasm, with transient ischemia reported in association with excessive blood loss [10,14].

#### 3.1.1. Popliteal Artery Thrombosis

Popliteal artery thrombosis (PAT) is the most common mechanism of iatrogenic injury among those previously mentioned, accounting for up to 60% of popliteal artery injuries following total knee arthroplasty (TKA) [15,16]. Popliteal artery damage may result from tourniquet use, which, while providing a bloodless field and improved visualization, can also interrupt the blood flow and promote stasis, all conditions that can facilitate arterial thrombosis, particularly in elderly patients with atherosclerosis. Additionally, PAT may result from traction and distortion of the popliteal artery during knee surgery, which can lead to plaque rupture and indirect vessel injury, thereby facilitating thrombus formation, particularly in non-compliant vessels [11,15].

Cardiovascular risk factors, such as diabetes, hypertension, smoking, history of arteriopathy, and advanced age, may contribute to PAT, placing these patients at higher risk of popliteal artery injury during knee surgery and warranting closer perioperative attention [17]. In such patients, the use of a tourniquet is still a matter of debate, and some authors advise against its use in patients with pre-existing peripheral vascular disease [18].

#### 3.1.2. Direct Arterial Injuries

Although uncommon, direct injuries to the popliteal artery during orthopaedic knee surgery represent serious complications, with prior studies indicating an associated major limb amputation risk of 47% [17].

Sharp injuries may occur during the posterior cut of the femoral condyles, tibial cuts, screw insertion for cementless tibial component stabilization, and bone cuts in general [11,19]. The risk is particularly high in “redo” orthopedic procedures, due to the presence of scar tissue and potential alterations in vascular anatomy, including the fixation of the artery closer to the knee joint within fibrotic tissue [2,13]. Additionally, cadaveric studies have shown that during total knee replacement surgeries, the steps of posterior retraction, hyperextension, hyperflexion and the exposed sharp bony edges after tibial osteotomy may contribute to the risk of the popliteal artery or collaterals injury [20].

Anatomical variability of the crural vessels, present in around 10% of cases, is another factor to be taken into account. These variations most often involve an arterial course situated closer to the periosteum and tibial cortex, or an unusually proximal bifurcation of the anterior and posterior tibial arteries. Given that such vascular anomalies are frequently asymptomatic, preoperative CT imaging is recommended for “redo” procedures or when an anatomical variation is suspected [21,22].

#### 3.1.3. Arteriovenous Fistulae

An arteriovenous fistula (AVF) is usually secondary to simultaneous incisions of an adjacent vein and artery [2]. In the context of knee surgery, iatrogenic AVFs typically involve the geniculate arteries, whereas reports of AVF formation between the popliteal vessels remain scarce [23,24]. The formation of an AVF typically results from a combination of factors, including tourniquet use, surgical instrumentation, and extremes of knee hyperextension or hyperflexion. These conditions may trigger inflammatory processes within the vasculature, leading to fistula development [25].

The clinical manifestations of AVF may include swelling, venous hypertension with varicosities, increased skin temperature, and alterations in skin pigmentation. A pulsatile mass accompanied by an audible bruit may also be observed, along with signs of digital ischemia or even gangrene [11]. Although most traumatic AVFs are diagnosed within the first week, some may present months or even years later, as patients often underestimate subtle symptoms [25].

#### 3.1.4. Pseudoaneurysm

The incidence of PA pseudoaneurysm secondary to knee surgery is reported to be from 0.03% to 0.17% [26].

An iatrogenic PA pseudoaneurysm is usually the result of a leak through all three layers of the vessel wall, which leads to the formation of hematoma that lies outside the blood vessels [2]. The underlying mechanism often involves direct or indirect arterial injury from surgical tools, thermal insult from bone cement, or cumulative damage from repeated local trauma [26,27]. The clinical presentation of these pseudoaneurysms depends on the size and extent of the associated hematoma. Some are symptomatic, manifesting as a painful and/or pulsatile swelling, whereas others may remain clinically silent when the pseudoaneurysmal sac is partially or completely thrombosed [2].

### 3.2. Orthopedic Knee Procedures Most Commonly Involved

The main orthopaedic knee procedures in which the popliteal artery may be injured include TKA, arthroscopic procedures, meniscus repair, hardware removal, and both posterior and anterior cruciate ligament reconstructions. A detailed overview of the mechanisms of injury, as well as precautions and recommendations, is provided in Table 1.

### 3.3. Diagnosis

In cases of iatrogenic popliteal artery injury following orthopedic knee surgery, early diagnosis and accurate assessment of the vascular damage are essential to improving outcomes and reducing the risk of potentially life-threatening complications. Nevertheless, these injuries may present with subtle or absent clinical signs, and up to 5–15% of individuals with vascular damage may exhibit a normal pulse on examination [2].

For practical purposes, the diagnostic approach can be divided into two main clinical scenarios: (1) patients with clear signs of acute vascular injury and (2) patients with equivocal or delayed presentations.

1. In the presence of overt clinical signs of vascular injury, manifesting as uncontrolled haemorrhage, rapidly increasing hematoma, and/or any of the typical indicators of limb ischemia (pain, pallor, pulselessness, paraesthesia, paralysis) [32], imaging studies such as computed tomography (CT) or conventional angiography are generally unnecessary, and an immediate vascular consult is recommended to avoid any delay in treatment.
2. In clinically equivocal cases, where the symptoms are less evident, or delayed, with the development of a pseudoaneurysm, arteriovenous fistulae or even partial incisions of the PA in the post-operative period [33,34], Doppler ultrasound may be useful for the initial assessment. However, computed tomography angiography (CTA) is considered the preferred diagnostic technique over other modalities, as it allows rapid and precise identification and localization of vascular injuries [2,34]. When the extent of the injury cannot be accurately evaluated on CTA, especially after TKA due to the presence of beam-hardening artifacts from the metallic endoprosthesis, digital subtraction angiography (DSA) may be helpful [21].

### 3.4. Treatment

The choice of treatment depends on the patient’s clinical condition and the severity of the vascular injury. As advised in the European Guidelines, every patient with suspected acute limb ischemia in a non-vascular centre should be transferred to a vascular centre that can provide the full spectrum of open and endovascular interventions [35].

#### 3.4.1. Treatment of Popliteal Artery Thrombosis (PAT)

Popliteal artery thrombosis can be treated with percutaneous thrombus aspiration, thrombo-embolectomy, urokinase with or without prostatcyclin [13,15]. These approaches are widely reported in the literature and are generally considered effective in restoring vessel patency, particularly when diagnosis is made early. It is worth considering that in patients undergoing orthopaedic knee surgery with secondary popliteal artery injuries, percutaneous approaches may be less invasive and have a smaller impact on rehabilitation compared to open surgery [15]. However, in cases in which intravascular recanalization cannot be achieved, usually in patients with acute or chronic ischaemia, surgical bypass can be considered. In such cases, a venous graft should be used, given the best patency rates of the latter compared to prosthetic grafts [36,37]. While minimally invasive approaches are increasingly adopted in clinical practice, their superiority over open surgery in terms of long-term functional outcomes remains insufficiently established.

#### 3.4.2. Treatment of Direct Arterial Injuries

According to previous reports, if a direct injury to the popliteal artery is suspected, the preferred approach should be a surgical exploration of the popliteal artery, especially in emergent or urgent cases, with hemodynamic instability or compartment syndrome [36]. For vascular reconstruction, most authors recommend using a reversed saphenous vein graft, whereas synthetic grafts (expanded polytetrafluoroethylene—ePTFE) have been employed less frequently. Other reported techniques include termino-terminal anastomosis, pinpoint suturing of the injured vessel, and venous patch repair [28,29,33,38]. In such cases, the posterior popliteal approach was most employed, as it provides better exposure of the popliteal neurovascular structures. Among incision techniques, the transverse incision was generally preferred over the S-shaped alternative [39,40]. If acute limb ischemia is complicated by compartment syndrome, fasciotomy should be performed as part of the intervention to prevent ischemic and neurological functional sequelae [33,37].

In selected cases, such as severe bleeding or in elderly patients, an endovascular approach with stent graft implantation may be considered. However, the supporting evidence for this approach remains limited, and concerns persist regarding long-term patency, especially in the highly mobile popliteal segment. These limitations are particularly relevant in younger orthopedic patients, in whom durability over time is a critical consideration. Therefore, while endovascular techniques may be appropriate in selected scenarios, their role as a primary treatment strategy remains to be clearly defined [21].

#### 3.4.3. Treatment of Popliteal Artery Pseudoaneuryms

As for direct arterial injuries and PAT, popliteal artery pseudoaneurysm can be treated either surgically or through endovascular techniques.

Traditionally, open repair with vein bypass has been the most commonly reported approach and remains widely accepted. In recent years, however, endovascular repair with covered stent grafts has emerged as a valid alternative, offering a lower risk of injury to adjacent structures and prosthetic infection [41]. Recent case series have reported satisfactory patency rates at one-year follow-up [42,43].

A hybrid approach may also be considered, as described by Vassiliou et al., in which a stent graft was initially deployed at the site of injury to control bleeding and seal the leakage. Subsequently, surgery was safely performed by clamping the artery proximally and distally, opening the sac, removing the stent graft, and extending to a longitudinal arteriotomy [44].

Another less invasive treatment option that has been documented in literature is ultrasound-guided compression or thrombin injection, aimed at inducing thrombosis. Although this technique has been reported in the literature with some favourable outcomes, the evidence is still limited to a few cases and has not yet been standardized [31,34].

#### 3.4.4. Treatment of Arteriovenous Fistulae

Given the rarity of this complication, evidence on the management of arteriovenous fistulas remains limited.

A 2024 review including 11 cases reported that 3 were managed with endovascular techniques, specifically with the interposition of a self-expanding Viabahn^®^ stent graft, whose flexibility and durability provided acceptable long-term patency rates [45]. The remaining cases underwent open surgery, with repair of both the popliteal vein and artery using vein grafts and bovine pericardium [46]. Due to the limited number of reported cases and the absence of comparative studies, no clear consensus can be established regarding the preferred treatment strategy. Management should therefore be individualized, taking into account patient characteristics, lesion type, and local expertise.

## 4. Discussion

Popliteal artery injury represents an uncommon but potentially devastating complication of orthopedic knee surgery. The present review highlights three clinically relevant aspects. First, the spectrum of vascular injury is broad and includes thrombosis, direct arterial incision, pseudoaneurysm formation, and arteriovenous fistulae, with clinical presentation ranging from acute limb-threatening ischemia or hemorrhage to subtle and delayed manifestations. Second, early recognition remains a major determinant of outcome, since diagnostic delay may result in prolonged ischemic time and consequently increase the risk of irreversible neurological damage and limb loss. Third, both open and endovascular treatment strategies are currently available, although the optimal approach remains dependent on the mechanism of injury, clinical presentation, patient characteristics, and institutional expertise.

An important finding emerging from the available literature is that the rarity of these complications should not lead to underestimation of their clinical relevance. In fact, the close anatomical relationship between the popliteal artery and the posterior knee structures creates a predictable anatomical vulnerability during several orthopedic procedures, particularly total knee arthroplasty, ligament reconstruction, osteotomy, and posterior instrumentation. Consequently, prevention, early suspicion, and prompt vascular assessment may be more relevant to prognosis than the choice of a specific revascularization technique itself.

### 4.1. Current Clinical Controversies

Despite increasing awareness of popliteal artery injuries associated with orthopedic knee surgery, several aspects of their diagnosis, treatment, and prevention remain controversial. From a diagnostic perspective, preserved distal pulses do not completely exclude significant arterial injury, while postoperative pain and swelling may mask subtle or delayed presentations. In patients with overt hemorrhage or acute limb ischemia, imaging should not delay vascular intervention, whereas CTA represents the preferred modality in clinically stable patients with equivocal findings; however, metallic artifacts following total knee arthroplasty may limit its accuracy, making digital subtraction angiography useful in selected cases [21,34]. Similar challenges have recently been highlighted by Asensio et al. in the context of traumatic popliteal vessel injuries, particularly regarding the importance of early recognition, minimization of ischemic time, and timely restoration of arterial flow [47]. Although traumatic and iatrogenic injuries represent distinct clinical scenarios, these principles remain relevant in orthopedic surgery, where delayed recognition may similarly compromise limb viability.

The optimal treatment strategy also remains debated. Although open reconstruction represents an established approach for extensive arterial damage or failed recanalization, endovascular techniques may reduce surgical trauma and facilitate postoperative rehabilitation. Nevertheless, concerns remain regarding long-term stent-graft durability in the highly mobile popliteal segment, and the available evidence is insufficient to establish the superiority of either approach [35,45]. Finally, prevention and perioperative risk stratification remain areas of uncertainty. Particular attention should be paid to patients with pre-existing vascular disease and to procedures involving the posterior knee, while the role of tourniquet use and routine preoperative vascular imaging remains debated. Rather than systematic vascular imaging in all patients, a selective approach based on individual vascular risk, abnormal clinical findings, previous knee surgery, or suspected anatomical variants appears more appropriate. Overall, these controversies highlight the need for individualized decision-making and closer collaboration between orthopedic and vascular specialists, particularly in high-risk or diagnostically uncertain cases.

### 4.2. Medico-Legal Appraisal Under Italian Civil Healthcare Liability Law

The analysis below concerns Italian civil healthcare liability. Under Law No. 24/2017, the applicable civil regime differs according to whether the claim is directed against the healthcare facility or an individual clinician and according to the legal relationship involved; this distinction must be made before evidentiary burdens are allocated [48,49]. Article 5 of the same law also requires attention to applicable guidelines and good clinical practice while preserving the specific features of the individual case [48]. The discussion therefore applies general Italian civil liability principles to the clinical circumstances of PAI; it does not propose a PAI-specific legal test or an exhaustive synthesis of Italian PAI case law.

#### 4.2.1. Professional Standard and Ex-Ante Risk Recognizability

PAI is a known but uncommon complication of knee surgery. The literature on posterior knee anatomy, vascular disease, revision surgery, anatomical variants, and procedure-specific mechanisms provides the clinical background for risk assessment [21,33,50], but it does not by itself show that a particular injury was foreseeable, avoidable, or negligently caused. Italian case law likewise does not treat the clinical label “complication” as an independently exculpatory category [51]. The assessment should instead begin with what was reasonably knowable in the individual case: the patient’s vascular history and examination, previous surgery, available imaging or anatomical information, and the specific procedural step involved. It should then consider whether a clinically justified precaution was applicable and whether it was actually adopted. These are separate questions and should not be conflated with the later issue of causation.

The targeted search found little literature specifically addressing the medico-legal consequences of PAI. Parvizi et al. reported lawsuits following vascular injuries after total joint arthroplasty, while Bhutta et al. described a claim involving popliteal-vessel injury followed by above-knee amputation [52,53]. Earlier medico-legal literature also recognized the potential legal consequences of unrecognized or improperly managed popliteal vascular injury in orthopedic surgery [54]. Broader orthopedic evidence identifies diagnostic and procedural errors as recurrent malpractice allegations [55], while recent consent literature documents persistent medico-legal and documentation problems in elective orthopedic surgery [56]. These foreign data provide context only; they do not define Italian negligence, burden of proof, or causation standards.

#### 4.2.2. Delayed Recognition, Causation, and Additional Harm

Two questions should be considered separately: whether the conduct at issue caused the primary arterial injury and whether a later failure to recognize, investigate, escalate, or treat vascular compromise worsened its consequences. A postoperative interval is not, by itself, a negligent delay. The reconstruction should distinguish biological onset, the first symptoms or patient report, documented clinical recognition, the point at which vascular compromise should reasonably have been recognized, specialist consultation or imaging, and definitive vascular treatment. This is particularly important because preserved distal pulses and initially equivocal symptoms may complicate early recognition [2,21].

Causation must then be tested against a realistic alternative course of care. In contractual healthcare liability, Cassation judgment No. 28991/2019 requires the claimant to establish the material causal nexus between the healthcare conduct and the new injury or worsening before the burden concerning exact performance or a non-imputable impediment becomes relevant [49]. For an alleged omission, Cassation order No. 16199/2024 requires a case-specific assessment of whether the omitted conduct would have prevented the harmful event, considering the evidence as a whole and alternative explanations rather than relying on population frequencies alone [57]. Where the allegation concerns delayed recognition or rescue, the relevant comparator is ordinarily the same primary vascular injury followed by timely and feasible management, not an entirely uninjured limb. The question is therefore whether appropriate recognition and rescue would, more likely than not, have prevented or reduced the additional ischemic, neurological, or limb-loss consequences. The severity of the final outcome does not replace proof of additional harm attributable to the delay.

#### 4.2.3. Informed Consent

Informed consent requires separate consideration. Article 1 of Law No. 219/2017 requires information that is complete, updated, and comprehensible regarding diagnosis, prognosis, benefits and risks of the proposed treatment, reasonable alternatives, and the consequences of refusal, together with appropriate documentation of the consent process [58]. Italian case law further indicates that low statistical frequency does not, by itself, remove a foreseeable risk from the informational duty, while absolutely exceptional or highly improbable events need not be listed indiscriminately [59]. This does not create a universal PAI disclosure threshold: the level of detail required must be assessed in the context of the actual clinical decision and the information relevant to that patient and procedure.

A signed form documents the consent process but is not a substitute for adequate information, and disclosure of a complication does not establish compliance with the technical standard of care. Conversely, deficient information and technically negligent treatment are distinct alleged breaches. Cassation judgment No. 28985/2019 emphasizes the autonomous protection of patient self-determination and the need to distinguish consent-related harm from injury caused by the healthcare treatment itself [60]. The appraisal should therefore identify what information was owed, what was actually communicated, the nature of the alleged informational deficiency, and the specific consequence attributed to it.

#### 4.2.4. Documentation and Evidentiary Reconstruction

Clinical documentation is particularly relevant because it allows the care pathway to be reconstructed retrospectively. Article 26 of the Italian Code of Medical Deontology requires the clinical record to be complete, clear, diligent, and capable of ensuring traceability of the clinical course; Articles 33 and 35 address patient information and consent [61]. In a PAI case, relevant material may include the preoperative vascular assessment, operative and anesthetic records, intraoperative events, serial neurovascular findings, nursing observations, symptom reports, imaging timestamps, vascular consultation or transfer records, and findings at revascularization. Absence of an entry is not automatically proof that an action was omitted. Cassation order No. 16737/2024 recognizes that incomplete records may have evidentiary consequences when the incompleteness itself prevents causal reconstruction and the challenged conduct is otherwise capable of producing the damage [62].

The resulting appraisal questions are summarized in Table 2.

### 4.3. Methodological Limitations and Potential Risk of Bias

The available evidence on popliteal artery injury following orthopedic knee surgery is characterized by substantial methodological heterogeneity. A considerable proportion of the literature consists of case reports and small case series, which are inherently prone to selection and publication bias and do not allow reliable estimation of incidence, comparative effectiveness, or causal relationships. Larger observational studies provide more robust estimates but remain susceptible to retrospective data collection, coding or classification errors, incomplete ascertainment of events, and residual confounding. In addition, differences in patient selection, type of orthopedic procedure, definition of vascular injury, treatment strategies, and duration of follow-up contribute to clinical heterogeneity across studies. These limitations should therefore be considered when interpreting the findings of the present narrative review and preclude direct conclusions regarding the superiority of specific diagnostic or therapeutic strategies.

The medico-legal component is intentionally limited to Italian civil healthcare liability and applies general legal authorities to PAI-specific clinical evidence; it is not an exhaustive review of Italian PAI case law. Foreign litigation studies were used only descriptively. The targeted medico-legal searches were designed for narrative source identification, not as a systematic review of all legal literature.

## 5. Conclusions

Although uncommon, popliteal artery injury remains a potentially limb-threatening complication of orthopedic knee surgery. Prompt recognition, thorough knowledge of posterior knee anatomy, and timely referral to a vascular specialist are essential to minimize ischemic time and improve limb salvage. Given the limited evidence available, treatment should be individualized according to the mechanism of injury, clinical presentation, patient characteristics, and local expertise. From an Italian civil medico-legal perspective, the occurrence of PAI neither establishes negligence nor excludes it merely because the event is a recognized complication. Appraisal requires case-specific assessment of the professional standard, the alleged departure from that standard, and the causal nexus with the claimed harm. When delay is alleged, the primary vascular event should be distinguished from any additional harm that timely and feasible rescue would, more likely than not, have prevented or reduced. Informed consent and clinical documentation are separate domains and do not substitute for assessment of technical conduct and causation.

## Figures and Tables

**Table 1:** Overview of orthopedic knee procedures associated with popliteal artery injury, including the main mechanisms of vascular damage and recommended precautions and preventive measures for orthopedic surgeons.

Orthopedic Procedure	Mechanism of Popliteal Artery Injury	Precautions and Recommendations for Orthopedic Surgeons
**Anterior Cruciate Ligament Reconstruction (ACL-R)**	Injury may occur during tibial tunnel drilling, from misplaced fixation devices (e.g., interference screws or buttons), or during posterior retraction. The close proximity of the artery to the posterior tibial cortex increases the risk [28,29]	A thorough understanding of posterior knee anatomy is essential. Excessive drilling depth should be avoided, fluoroscopic guidance may be considered when necessary, and the posterior capsule should be adequately protected during tibial tunnel creation.
**Posterior Cruciate Ligament Reconstruction (PCL-R)**	This procedure carries a higher risk due to the close anatomical relationship between the PCL tibial insertion and the popliteal artery (3–16 mm). Injuries have been reported during posteromedial portal creation, tibial drilling, or graft passage [28,29]	The tibial tunnel should be performed with the knee flexed at approximately 90°. The use of protective techniques, such as a balloon catheter spacer, may be considered. Posterior dissection should be minimized, and vascular surgical support should be readily available.
**Arthroscopic Meniscus Root Repair (esp. lateral root)**	Popliteal artery pseudoaneurysm may result from penetration of suture anchors or needles through the posterior horn into the artery [30].	Controlled-depth drilling is recommended. Posterior safety portals should be used only when strictly necessary, and particular caution is required when placing anchors near the posterior horn.
**Total Knee Arthroplasty (TKA)**	Injury may occur due to direct incision during tibial or femoral cuts, retractor placement, or thermal injury from bone cement. Arterial occlusion may also result from thrombosis related to tourniquet use. Revision surgery represents an additional risk factor due to scar tissue and altered anatomy [26,31]	The knee should be maintained in flexion to maximize the distance between the artery and the tibia. Excessive posterior retraction should be avoided, and caution is required during cement application. If vascular injury is suspected, the tourniquet should be promptly released and immediate vascular consultation obtained.
**Hardware Removal (distal femur/tibia)**	Although rare, pseudoaneurysm formation may occur following removal of screws or plates, particularly in patients with connective tissue disorders (e.g., Marfan syndrome). Delayed presentation is possible [4].	Preoperative vascular risk assessment is recommended in high-risk patients. Careful dissection should be performed during hardware removal, and postoperative monitoring for hematoma or pulsatile masses is essential.
**Tibial Osteotomy**	During osteotomy of the lateral compartment, the popliteal artery is often located posterior to the osteotomy plane and in close proximity to the posterior tibial cortex, both in flexion and extension. In a minority of medial compartment cases, a similar relationship may be observed [6]	Accurate intraoperative identification of the arterial position is required. Careful surgical technique and postoperative monitoring for signs of vascular injury are recommended.
**Femoral Osteotomy**	No anatomical structure separates the distal femur from the popliteal artery. The use of a tourniquet may obscure intraoperative signs of vascular injury, and the medial approach is associated with a higher risk [6].	Preoperative assessment of arterial location in relation to the surgical approach is essential. Increased caution is required during medial osteotomy compared to lateral approaches.

**Table 2:** Operational medico-legal framework for appraisal of popliteal artery injury under Italian civil healthcare liability law.

Medico-Legal Issue	Applicable Legal/Forensic Criterion	PAI-Specific Appraisal Question	Evidence Required for Reconstruction
**Applicable liability regime and burden of proof**	Identify the defendant and legal relationship before allocating evidentiary burdens. In contractual healthcare liability, material causation and performance/non-imputability are distinct proof issues [48,49].	Is the claim directed at the healthcare facility and/or an individual clinician, and which disputed proposition must each party establish under the applicable regime?	Event date; organizational and contractual relationships; allocation of responsibilities; pleadings and expert questions; relevant contemporaneous records.
**Ex-ante risk recognizability and professional standard**	Assess the conduct reasonably required on the information available at the time, considering applicable guidelines or good clinical practice and the specific features of the case [48].	What vascular risk was reasonably recognizable for this patient and procedure, and what precaution was clinically justified at that time?	Vascular history and examination; previous surgery; available imaging/anatomical information; procedure and operative plan; applicable recommendations and versions.
**Alleged breach and primary vascular injury**	Distinguish the clinical mechanism of injury from a proven departure from required conduct; the label “complication” neither establishes nor excludes responsibility [51].	What specific act or omission is alleged to have caused the laceration, thrombosis, pseudoaneurysm, or other arterial lesion?	Operative and anesthetic records; instruments and technique; intraoperative findings; lesion morphology; vascular exploration or repair findings; competing explanations.
**Recognition and escalation**	Distinguish delayed clinical presentation from an unjustified diagnostic or treatment delay and identify when a competent response was reasonably required.	When did vascular compromise become reasonably recognizable, and were examination, imaging, vascular consultation, transfer, and treatment escalated appropriately?	Serial neurovascular findings; symptom reports; nursing/anesthetic records; timestamps for imaging, consultation, transfer, and intervention.
**Causation and additional harm**	Apply the civil causal standard to a specified, feasible counterfactual and distinguish the primary injury from later aggravation [49,57].	Would timely and feasible recognition and rescue, more likely than not, have prevented or reduced the claimed ischemic, neurological, or limb-loss consequences?	Lesion mechanism; perfusion and tissue viability; treatment feasibility; ischemic time; alternative causes; expected course with timely rescue; actual sequelae.
**Informed consent**	Assess the information owed and supplied separately from technical performance; low frequency alone does not remove a foreseeable risk from the informational duty [58,59,60].	What information was relevant to the actual surgical decision, what was communicated, and what specific consent-related consequence is alleged?	Consent discussion and documents; procedure-specific risk information; alternatives; patient questions and preferences; emergency circumstances where relevant.
**Documentation and evidentiary reconstruction**	Clinical records should be complete and traceable; incomplete documentation has conditional, not automatic, evidentiary significance [61,62].	Does missing or inconsistent documentation prevent reconstruction of a causally relevant event, and can other contemporaneous evidence resolve the gap?	Complete chart; operative, anesthetic, and nursing records; imaging systems; consultation and transfer records; timing discrepancies; other contemporaneous evidence.

Note: This framework is an author-developed appraisal aid, not a validated scoring instrument. The evidence listed is case-dependent and does not imply that every investigation, precaution, or consultation is mandatory in every patient.

## Data Availability

No new data were created or analyzed in this study. Data sharing is not applicable to this article.

## Supplementary Materials

The following supporting information can be downloaded at: https://www.mdpi.com/article/10.3390/diseases14090335/s1, Table S1. Medico-legal database search strategies and raw yields.

## Abbreviations

The following abbreviations are used in this manuscript:

ACL	Anterior Cruciate Ligament
ACL-R	Anterior Cruciate Ligament Reconstruction
ATA	Anterior Tibial Artery
AVF	Arteriovenous Fistula
CTA	Computed Tomography Angiography
CT	Computed Tomography
DSA	Digital Subtraction Angiography
DVT	Deep Vein Thrombosis
ePTFE	Expanded Polytetrafluoroethylene
ESVS	European Society for Vascular Surgery
MeSH	Medical Subject Headings
PA	Popliteal Artery
PAI	Popliteal Artery Injury
PAT	Popliteal Artery Thrombosis
PCL	Posterior Cruciate Ligament
PCL-R	Posterior Cruciate Ligament Reconstruction
PEA	Peroneal Artery
PTA	Posterior Tibial Artery
TKA	Total Knee Arthroplasty
