# Supplementary material for: Popliteal Artery Injuries in Orthopedic Knee Surgery: A Narrative Review with an Italian Medico-Legal Perspective
Source: Diseases. 2026 Sep 13;14(9):335. doi: 10.3390/diseases14090335 (PMC13605625; doi:10.3390/diseases14090335)
Supplement: Supplementary file 1 [file diseases-14-00335-s001.zip › diseases-4499972-supplementary.pdf]

Supplementary Table S1. Medico-legal database search strategies and raw yields

All searches were executed on 7 September 2026. Counts are raw database yields before deduplication. The first tier was designed to identify PAI/knee-surgery-specific medico-legal evidence; the second tier was intentionally broader and was used for contextual source identification within the narrative review design.

ID	Database	Purpose	Raw yield
PM-A	PubMed	PAI/knee-surgery-specific medico-legal search	9
SC-A	Scopus	PAI/knee-surgery-specific medico-legal search	14
WS-A	Web of Science Core Collection	PAI/knee-surgery-specific medico-legal search	9
PM-B	PubMed	Broader orthopedic/vascular contextual medico-legal search	20
SC-B	Scopus	Broader orthopedic/vascular contextual medico-legal search	1,175
WS-B	Web of Science Core Collection	Broader orthopedic/vascular contextual medico-legal search	429

Exact search strategies

PM-A — PubMed

```
(
  ("Popliteal Artery"[Mesh] OR "popliteal arter*"[Title/Abstract] OR "popliteal vessel*"[Title/Abstract])
  AND
  ("Knee Joint/surgery"[Mesh] OR "Arthroplasty, Replacement, Knee"[Mesh] OR "Arthroscopy"[Mesh]
   OR "knee surg*"[Title/Abstract] OR "total knee arthroplast*"[Title/Abstract]
   OR "knee arthroscop*"[Title/Abstract] OR "cruciate ligament reconstruction"[Title/Abstract]
   OR "tibial osteotom*"[Title/Abstract])
  AND
  ("Malpractice"[Mesh] OR "Liability, Legal"[Mesh] OR "Jurisprudence"[Mesh] OR "Informed Consent"[Mesh]
   OR medicolegal[Title/Abstract] OR "medico-legal"[Title/Abstract]
   OR malpractice[Title/Abstract] OR litigation[Title/Abstract] OR liability[Title/Abstract]
   OR negligence[Title/Abstract] OR "standard of care"[Title/Abstract]
   OR "informed consent"[Title/Abstract] OR "diagnostic delay"[Title/Abstract]
   OR "delayed diagnosis"[Title/Abstract] OR "treatment delay"[Title/Abstract])
)
```

SC-A — Scopus

```
TITLE-ABS-KEY(
  ("popliteal arter*" OR "popliteal vessel*")
  AND
  ("knee surg*" OR "total knee arthroplast*" OR "knee arthroscop*"
   OR "cruciate ligament reconstruction" OR "tibial osteotom*")
  AND
  (medicolegal OR "medico-legal" OR malpractice OR litigation OR liability
   OR negligence OR "standard of care" OR "informed consent"
   OR "diagnostic delay" OR "delayed diagnosis" OR "treatment delay")
)
```

WS-A — Web of Science Core Collection

```
TS=(
  ("popliteal arter*" OR "popliteal vessel*")
  AND
  ("knee surg*" OR "total knee arthroplast*" OR "knee arthroscop*"
   OR "cruciate ligament reconstruction" OR "tibial osteotom*")
  AND
  (medicolegal OR "medico-legal" OR malpractice OR litigation OR liability
   OR negligence OR "standard of care" OR "informed consent"
   OR "diagnostic delay" OR "delayed diagnosis" OR "treatment delay")
)
```

PM-B — PubMed

```
(
  (
    "Orthopedic Procedures"[Mesh]
    OR "Arthroplasty, Replacement, Knee"[Mesh]
    OR "Knee Joint/surgery"[Mesh]
    OR "Arthroscopy"[Mesh]
    OR orthopaedic surg*[Title/Abstract]
    OR orthopedic surg*[Title/Abstract]
    OR knee surg*[Title/Abstract]
    OR total knee arthroplast*[Title/Abstract]
    OR knee arthroscop*[Title/Abstract]
  )
  AND
  (
    (
      "Vascular System Injuries"[Mesh]
      OR vascular injur*[Title/Abstract]
      OR arterial injur*[Title/Abstract]
      OR vascular complication*[Title/Abstract]
    )
    AND
    (
      "Malpractice"[Mesh]
      OR "Liability, Legal"[Mesh]
      OR medicolegal[Title/Abstract]
      OR "medico-legal"[Title/Abstract]
      OR malpractice[Title/Abstract]
      OR litigation[Title/Abstract]
      OR liability[Title/Abstract]
      OR negligence[Title/Abstract]
      OR "standard of care"[Title/Abstract]
      OR "diagnostic delay"[Title/Abstract]
      OR "delayed diagnosis"[Title/Abstract]
      OR "treatment delay"[Title/Abstract]
    )
  )
  OR
  (
    (
      "Orthopedic Procedures"[Mesh]
      OR "Arthroplasty, Replacement, Knee"[Mesh]
      OR orthopaedic surg*[Title/Abstract]
      OR orthopedic surg*[Title/Abstract]
      OR knee surg*[Title/Abstract]
      OR total knee arthroplast*[Title/Abstract]
    )
    AND
    (
      "Informed Consent"[Mesh]
      OR "informed consent"[Title/Abstract]
      OR "consent documentation"[Title/Abstract]
    )
  )
)
```

SC-B — Scopus

```
TITLE-ABS-KEY(
  (
    ("orthopaedic surg*" OR "orthopedic surg*" OR "knee surg*"
     OR "total knee arthroplast*" OR "knee arthroscop*")
    AND
    (
      (
        ("vascular injur*" OR "arterial injur*" OR "vascular complication*")
        AND
        (medicolegal OR "medico-legal" OR malpractice OR litigation
         OR liability OR negligence OR "standard of care"
         OR "diagnostic delay" OR "delayed diagnosis" OR "treatment delay")
      )
      OR
      ("informed consent" OR "consent documentation")
    )
  )
)
```

WS-B — Web of Science Core Collection

```
TS=(
  (
    ("orthopaedic surg*" OR "orthopedic surg*" OR "knee surg*"
     OR "total knee arthroplast*" OR "knee arthroscop*")
    AND
    (
      (
        ("vascular injur*" OR "arterial injur*" OR "vascular complication*")
        AND
        (medicolegal OR "medico-legal" OR malpractice OR litigation
         OR liability OR negligence OR "standard of care"
         OR "diagnostic delay" OR "delayed diagnosis" OR "treatment delay")
      )
      OR
      ("informed consent" OR "consent documentation")
    )
  )
)
```

Note: The broader search was intentionally sensitive and was not treated as a systematically screened litigation corpus. Foreign empirical sources were used only for contextual evidence and not to define Italian legal standards.
